# Comparison between Vacuum-Assisted Closure Technique and Conventional Approach in Patients with Mediastinitis After Isolated Coronary Artery Bypass Graft Surgery

**DOI:** 10.21470/1678-9741-2022-0317

**Published:** 2023

**Authors:** Hakan Akbayrak, Hayrettin Tekumit

**Affiliations:** 1 Department of Cardiovascular Surgery, Selçuk University Faculty of Medicine, Konya, Turkiye; 2 Department of Cardiovascular Surgery, Bandırma Onyedi Eylul University Faculty of Medicine, Balıkesir, Turkiye

**Keywords:** Coronary Artery Bypass, Mediastinitis, Treatment, Vacuum-Assisted Closure

## Abstract

**Introduction:**

Median sternotomy is the most preferred approach in heart surgery. Post-sternotomy mediastinitis is a catastrophic and potentially life-threatening complication with an incidence rate of 0.15% to 5%, and its overall mortality rate reaches 47%. In this study, we aimed to compare the results of vacuum-assisted closure technique and the conventional methods on the management of mediastinitis following isolated coronary artery bypass graft surgery.

**Methods:**

Between February 2001 and July 2013, 32,106 patients who underwent cardiac operations were evaluated retrospectively. One hundred and fourteen patients who developed post-sternotomy mediastinitis were included in this study. The patients were divided into two groups and compared - vacuum-assisted closure group (n=52, 45.6%) and conventional treatment group (n=62, 54.4%).

**Results:**

There were no differences between the two groups according to the patients’ characteristics, surgical data, and mediastinal cultures. However, we found that total treatment duration for post-sternotomy mediastinitis, time interval from diagnosis to negative culture, hospitalization time, and in-hospital mortality were statistically significantly lower in the vacuum-assisted closure group than in the conventional treatment group (P<0.001, P<0.001, P<0.001, and P=0.03, respectively).

**Conclusion:**

This study demonstrates that the vacuum-assisted closure technique improves the medical outcome of patients with post-sternotomy mediastinitis compared with the conventional treatment. The vacuum-assisted closure is a safe and more effective treatment modality for patients with post-sternotomy mediastinitis after cardiac surgery with reasonable morbidity and mortality.

## INTRODUCTION

The idea of using median sternotomy as an approach to the thoracic organs came up in the late 1800s^[1]^. Although minimally invasive techniques have gained popularity in recent years, median sternotomy remains the most common approach for heart surgery. Post-sternotomy mediastinitis (PSM), particularly following coronary artery bypass graft surgery, is a catastrophic and potentially life-threatening complication^[2,3]^. Despite the fact that it is an uncommon complication with an incidence rate of 0.15% to 5%, its overall mortality rate reaches 47%^[1-5]^.

Chest pain, sternal dehiscence, fever, purulent discharge, and/or isolation of microorganisms in mediastinal drainage cultures are among the diagnostic criteria for PSM^[6]^. In the development of a deep sternal wound infection (DSWI), sternal instability is the critical event. It is followed by skin degeneration and microbial leakage into the deeper tissues. The alternative scenario for mediastinitis pathogenesis is insufficient mediastinal drainage, which results in a substantial retrosternal collection that acts as a bacterial culture^[1]^.

Risk factors for mediastinitis can be classified into three categories: patient-related, intraoperative, and postoperative. Risk factors associated with patients include older age, obesity, smoking, and the presence of concomitant conditions such as diabetes mellitus and/or chronic lung disease. Chronic infections (*e.g.*, human immunodeficiency virus, hepatitis B or C virus, or bacterial infections lasting more than four weeks) also are risk factors for DSWI^[7]^.

Sterile wound dehiscence occurs more frequently than DSWI. The sterile wound dehiscence occurred in 60% of patients who had a wound complication after median sternotomy^[8]^. Although predisposing risk factors for sterile wound dehiscence and DSWI are similar, treatment approaches are different. The most commonly isolated microorganisms in PSM are Gram-positive bacteria. *Staphylococcus aureus* or *Staphylococcus epidermidis* are responsible for 70 to 80% of cases^[1,4,9]^.

Vacuum-assisted closure (VAC) is a relatively novel breakthrough in wound care which has begun to replace conventional methods. Some studies have concluded that VAC is a safe and effective treatment option in PSM when compared to conventional treatment (CoT)^[3,4]^. Recently, despite the improvement of sterilization techniques and the modern operating room designs, the evidence for standard management of DSWI after cardiac surgery is still controversial.

In this retrospective study, we aimed to compare the results of VAC technique and the CoT on the management of mediastinitis following isolated coronary artery bypass graft surgery.

## METHODS

**Fig. 1:**
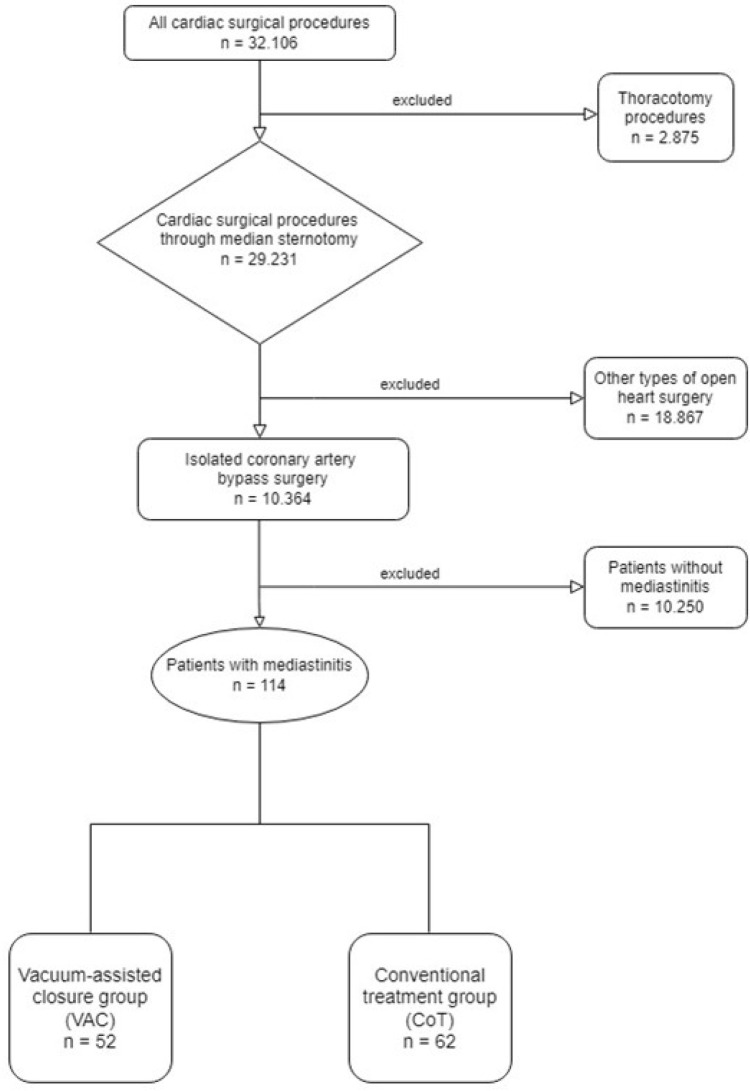
Flow chart of the study design.

All cardiac operations including 32,106 procedures between February 2001 and July 2013 were evaluated retrospectively. Thoracotomy procedures, operations other than isolated coronary artery bypass graft surgery, and patients with non-microbial sternal dehiscence were excluded from the study. A total of 10,364 isolated coronary artery bypass graft operations via median sternotomy were analyzed, and 114 (1.1%) patients were found to develop mediastinitis postoperatively. The patients who developed PSM were divided into two groups - VAC group (n=54, 45.6%) and CoT group (n=62, 54.4%) (Figure 1).

Between February 2001 and December 2006, all PSM cases were managed with the CoT in our center. Since January 2007, all of the PSM patients were treated with the VAC technique. The diagnosis of PSM was based on at least one of the following criteria of the Centers for Disease Control and Prevention^[10]^: (1) isolation of microorganisms in the mediastinal drainage cultures; (2) evidence of mediastinitis seen during operation; and (3) sternal instability, fever (> 38°C), and/or purulent discharge from the mediastinum or isolation of microorganisms in the blood/mediastinal drainage cultures.

Our standard prophylactic antibiotic therapy was cefazolin sodium four times a day, at operative day and postoperative 1^st^ and 2^nd^ days. In tissue cultures from patients who were diagnosed with PSM, we usually started the antibiotic therapy with vancomycin hydrochloride intravenously two times a day when the Gram-positive microorganism was detected. Piperacillin-tazobactam combination was used for antibiotic therapy three times a day when the Gram-negative microorganism was detected. Antibiotic therapy was usually continued until tissue cultures results became available. Thereafter, the antibiotic therapy was adjusted according to bacterial sensitivity and strain.

When sternal infection was detected, firstly we opened the wound incision and removed the sternum wires of the PSM patients under aseptic conditions. Then, aggressive sternal and tissue debridement was performed in both groups. Then, we performed the procedures that included irrigation with povidone-iodine and saline solutions and open packing 3-4 times a day in the CoT group. We revised and rewired the sternum after three consecutive negative tissue cultures and as a result of the formation of a satisfactory granulation tissue in the wound in the CoT group.

All PSM patients in the VAC group underwent wound incision and removal of the sternum wires under aseptic conditions. Thereafter, aggressive sternal and tissue debridement was done. In this group, a VAC system, polyurethane foam, and a special computer-controlled pump unit were used. The polyurethane sponge was fitted into the wound substernally. The others were placed between the sternal edges and the subcutaneous layer, respectively. The wound was covered with an adhesive, semipermeable drape that was connected to the therapy unit. The therapy unit delivers a negative pressure between -75 mmHg and -150 mmHg in a continuous mode. We revised and rewired the sternum, after three consecutive negative tissue cultures and as a result of the formation of a satisfactory granulation tissue in the wound in VAC group as well.

### Statistical Analyses

Statistical analyses were performed using the IBM Corp. Released 2013, IBM SPSS Statistics for Windows, version 22.0, Armonk, NY: IBM Corp. Distribution of continuous variables was assessed with the Kolmogorov-Smirnov test. Continuous variables were expressed as mean ± standard deviation for normally distributed variables. Non-normally distributed continuous variables were expressed as median and minimum-maximum values. Nominal variables were given as number and percentage. Categorical variables were compared with the Chi-square test, and continuous variables were compared with Student’s *t*-test or Mann-Whitney U test. Paired samples *t*-test was used to compare repeated measures. *P*-value of < 0.05 was considered as statistically significant.

## RESULTS

There were no significant differences between the two groups according to the patients’ baseline characteristics (Table 1). The mean age of the patients was 68.4±8.9 years in the VAC group and 71.2±9.3 years in the CoT group (*P*=0.1). While 16 of the patients in the VAC group were female (30.77%) and 36 were male (69.23%), 18 of the patients were female (29.03%) and 44 were male (70.97%) in the CoT group. The number of patients with body mass index ≥ 30 in the VAC group was 17 (32.69%) and 23 (37.1%) in the CoT group.

**Table 1:** Baseline characteristics of the study population.

	All PSM patients	VAC group	CoT group	*P*-value
	(n=114)	(n=52)	(n=62)	
Age, years	69.9±9.2	68.4±8.9	71.2±9.3	0.1
Gender				ns
Male, n (%)	80 (70.18)	36 (69.23)	44 (70.97)	
Female, n (%)	34 (29.82)	16 (30.77)	18 (29.03)	
Diabetes mellitus, n (%)	54 (47.37)	25 (48)	29 (46.8)	ns
Hypertension, n (%)	58 (50.88)	28 (53.85)	30 (48.39)	0.69
BMI ≥ 30, n (%)	40 (35.09)	17 (32.69)	23 (37.1)	0.77
LVEF ≤ 30, n (%)	25 (21.93)	12 (23.07)	13 (20.97)	0.96
Chronic obstructive pulmonary disease, n (%)	28 (24.56)	13 (25)	15 (24.19)	ns
Renal dysfunction, n (%)	12 (10.53)	5 (9.61)	7 (11.29)	ns
Urgent/emergency operations, n (%)	10 (8.77)	5 (9.61)	5 (8.06)	ns
EuroSCORE value	7.97±3.39	8.3±3.5	7.7±3.3	0.35

BMI=body mass index; CoT=conventional treatment; EuroSCORE=European System for Cardiac Operative Risk Evaluation; LVEF=left ventricular ejection fraction; ns=not significant; PSM=post-sternotomy mediastinitis; VAC=vacuum-assisted closure

There were no statistically significant differences between the groups in terms of cardiopulmonary bypass time, cross-clamping time, total drainage amount, presence of redo operations, internal thoracic artery use, transfusion amount, and postoperative revision (Table 2).

**Table 2:** Operative characteristics of the study population.

	VAC group (n=52)	CoT group (n=62)	*P*-value
Cardiopulmonary bypass time, minutes	78.4±17.1	82.3±21.9	0.29
Cross-clamping time, minutes	66.3±14.1	70.2±16.8	0.18
Total amount of drainage, mL	550 (300-650)	520 (300-650)	ns
Reoperation, n (%)	3 (5.77)	4 (6.45)	ns
Harvested internal thoracic artery, n (%)	50 (96.15)	60 (96.77)	ns
Transfusion, mL	330 (0-400)	350 (0-400)	0.75
Postoperative revision, n (%)	3 (5.77)	4 (6.45)	ns

CoT=conventional treatment; ns=not significant; VAC=vacuum-assisted closure

Furthermore, both the C-reactive protein level and white blood cell count were comparable between VAC and CoT groups at the time of PSM diagnosis and at discharge after sternal closure (Table 3).

**Table 3:** C-reactive protein (CRP) level and white blood cell (WBC) count for both groups measured at the time of post-sternotomy mediastinitis (PSM) diagnosis and discharge.

		VAC group (n=52)		CoT group (n=62)		*P*-value
		mean±SD	min-max	mean±SD	min-max	
CRP (mg/L)	PSM diagnosis	95.21±38.12	70-149	98.74±41.94	72-156	0.64
	Discharge	21.02±13.37	18-40	24.61±12.83	21-43	0.15
WBC (×10^3^/mm^3^)	PSM diagnosis	20.62±3.87	15-24	19.51±4.12	13-23	0.14
	Discharge	5.09±2.67	4-dez.	5.94±2.23	3.8-11	0.07

CoT=conventional treatment; SD=standard deviation; VAC=vacuum-assisted closure

Culture-verified PSM pathogens were given in Table 4. *Staphylococcus* strains were the most common microorganisms in the microbiological examination and cultures. However, there was no significant difference between the two groups in terms of microbiological agents.

**Table 4:** Culture-verified post-sternotomy mediastinitis pathogens.

	VAC group (n=52)	CoT group (n=62)	*P*-value
Methicillin-sensitive *Staphylococcus aureus*, n (%)	33 (63.46)	41 (66.13)	0.92
*Staphylococcus epidermidis*, n (%)	11 (21.15)	13 (20.97)	ns
Methicillin-resistant *Staphylococcus aureus*, n (%)	5 (9.61)	6 (9.68)	ns
Others, n (%)	3 (5.7)	2 (3.23)	0.84

CoT=conventional treatment; ns=not significant; VAC=vacuum-assisted closure

When we compared the two groups using the El Oakley classification, we found no significant differences between them in terms of El Oakley PSM types (Table 5).

**Table 5:** Post-sternotomy mediastinitis according to El Oakley classification system.

	VAC group (n=52)	CoT group (n=62)	*P*-value
Type I, n (%)	5 (9.61)	4 (6.45)	0.78
Type II, n (%)	5 (9.61)	4 (6.45)	0.78
Type IIIA, n (%)	24 (46.15)	22 (35.48)	0.33
Type IIIB, n (%)	15 (28.85)	19 (30.64)	ns
Type IVA, n (%)	1 (1.92)	8 (12.9)	0.07
Type IVB, n (%)	-	3 (4.84)	0.31
Type V, n (%)	2 (3.85)	2 (3.23)	ns

CoT=conventional treatment; ns=not significant; VAC=vacuum-assisted closure

There was no significant difference between the two groups in time from the cardiac surgery to the diagnosis of PSM (*P*=0.31). However, total treatment duration, the time interval from diagnosis to negative culture, and hospital stay were significantly shorter in the VAC group than in the CoT group (*P*<0.001, *P*<0.001, and *P*<0.001, respectively). In-hospital mortality was lower in the VAC group (5.77%) than in the CoT group (20.97%; *P*=0.03) (Table 6).

**Table 6:** Post-sternotomy mediastinitis (PSM) associated characteristics of the study population.

	VAC group (n=52)		CoT group (n=62)		*P*-value
	mean±SD	min-max	mean±SD	min-max	
Time interval from cardiac operation to PSM diagnosis, days	13.24±8.31	mar.-71	15.37±13.87	mar.-97	0.31
Treatment duration, days	20.63±8.87	13-31	56.41±28.5	28-91	< 0.001
Time interval from diagnosis to negative culture, days	15.02±5.2	out.-28	33.27±10.91	21-51	< 0.001
Hospital stay, days	27.24±6.1	21-45	76.11±42.74	31-127	< 0.001
In-hospital mortality, n (%)	3 (5.77)		13 (20.97)		0.03

CoT=conventional treatment; SD=standard deviation; VAC=vacuum-assisted closure

There were significant differences between the two groups according to the surgical wound-healing procedures performed (Table 7). The number of sternal closure procedures with standard rewiring after wound-healing was significantly higher in the VAC group (94.23%) than in the CoT group (72.58%) (*P*=0.005). However, additional techniques for sternal closure such as pectoralis muscle flaps and omentoplasty, which are relatively complex procedures, after the wound-healing were higher in the CoT group than in the VAC group (*P*=0.015 for pectoral muscle flap). Vascularized tissue flaps (pectoralis flaps and omentoplasty) were performed by plastic surgeons when needed.

**Table 7:** Additional surgical wound-healing procedures for post-sternotomy mediastinitis.

	VAC group (n=52)	CoT group (n=62)	*P*-value
Standard rewiring, n (%)	49 (94.23)	45 (72.58)	0.005
Pectoral muscle flap, n (%)	3 (5.77)	15 (24.19)	0.015
Omentoplasty, n (%)	-	2 (3.23)	ns

CoT=conventional treatment; ns=not significant; VAC=vacuum-assisted closure

## DISCUSSION

Following heart surgery, infection of the sternotomy area is a potentially catastrophic and frequently fatal complication. According to previous studies, the incidence of postoperative mediastinitis ranges between 0.4 and 5%^[1,11]^. In our analysis, during an 11-year period, 10,364 isolated coronary artery bypass graft procedures via median sternotomy were analyzed, and 114 (1.1%) patients developed mediastinitis following surgery, which is consistent with the literature. Risk factors described in previous studies were also present in our patient population, such as: diabetes mellitus (47.37%), obesity (35.09%), chronic obstructive pulmonary disease (24.56%), renal dysfunction (10.53%), and urgent/emergency operation (8.77%). However, both VAC and CoT groups were similar in terms of baseline and operative characteristics, laboratory findings, culture-verified pathogens, and El Oakley classification.

A combination of surgical debridement and antibiotic therapy is required to treat mediastinitis. Systemic antibiotic therapy should be initiated as soon as a diagnosis of mediastinitis is confirmed or suspected and blood cultures are acquired. The antibiotic regimen should be revised immediately upon receipt of the results of blood and wound cultures. The cornerstone treatment for postoperative mediastinitis is surgical debridement.

At first, PSM was treated with surgical revision with multiple open dressing changes. After that, the treatment was completed by sternal rewiring or secondary healing. These treatment approaches were used for these patients for a long time. But the mortality rate was reported to be between 10 to 47% with this approach by various authors^[1-5,12]^. Thoracic instability, which is important for the healthy mechanical ventilation, was the major disadvantage of open dressings. The risk of other complications such as muscular weakening, thrombosis, and pneumonia increases because of the prolonged immobilization^[3]^. Bryant et al. developed continuous saline solution and antibiotic irrigation for PSM cases in 1969^[13]^. Although it is an important technique that offers a stable sternum, the reported mortality rates of this technique were high in previous studies^[14]^.

VAC is a relatively recent approach that comprises of an open-cell foam dressing covered with an adhesive drape. The dressing attached to a vacuum pump produces subatmospheric pressure continuously or intermittently. VAC permits to absorb exudate continuously with simultaneous thoracic stability and isolation of the wound. VAC therapy stimulates granulation tissue formation with an increased blood flow in the contiguous tissue^[15]^. A previous systematic review revealed that when negative pressure wound care was compared to various wound management techniques for PSM, it was related with clinical benefits such as shortened hospital stay, lower rates of reinfection, and decreased early mortality^[16]^. In our study, we found that total treatment duration for PSM, the time interval from diagnosis to negative culture, hospitalization time, and in-hospital mortality are statistically significantly lower in the VAC group when compared with the CoT group (*P*<0.001, *P*<0.001, *P*<0.001, and *P*=0.03, respectively).

The use of vascularized tissue flaps is another treatment modality for PSM patients. If a patient has severe soft tissue deficit, the flap may be the only option. Lee et al. described the technique of using omentum flap for sternal closure in 1976^[17]^. Besides, Jurkiewicz et al. initially described the using of pectoral flaps for sternal closure in 1980^[18]^. In our study, 20 patients underwent flap procedures. The need for pectoral muscle flap is significantly higher in the CoT group than in the VAC group (*P*=0.015). The VAC group, on the other hand, had significantly more standard wiring for sternum closure (*P*=0.005). According to the result of our study, VAC treatment has reduced the need for relatively sophisticated interventions to close the sternum in PSM patients compared to conventional methods, allowing simpler and cheaper techniques to be enough.

Since PSM is one of the most feared complications after cardiac surgery, it is crucial to ensure effective collaboration between each member of the multidisciplinary team, which includes cardiothoracic surgeons, plastic surgeons, intensivists, infectious disease specialists, and clinical microbiologists. The best surgical technique for mediastinitis after open-heart surgery is still a matter of debate. Because of its safety and reliability, VAC therapy has been routinely utilized to treat PSM in most of the clinics, and its usage in cardiac surgery seems to be increasing. Although there are numerous studies on the results of VAC therapy in the literature, our study has the advantages of including a large number of patients over a long period of time from a big volume center and demonstrating a comparable outcome of VAC treatment *vs.* traditional approaches.

### Limitations

The retrospective design of our study is the major limitation. On the other hand, the heterogeneity due to differences between protocols, level of surgeons’ experience, and treatment approach across surgical teams could have influenced our results. Another limitation of our study is that some patients were possibly missed because they had to come to our center from other cities to undergo surgery. Postoperative mediastinitis may have developed in these patients, and they may have been treated in the city where they live in. Lastly, we were not able to assess the long-term outcome of the patients. A blinded, prospective, randomized, multicenter study is required to corroborate our findings.

## CONCLUSION

In conclusion, our retrospective analysis could demonstrate that the VAC technique improves the medical outcome of patients with PSM compared with the CoT. VAC is a safe and more effective treatment modality for patients with PSM after cardiac surgery with reasonable morbidity and mortality.
